# The Application of Chemometrics in Metabolomic and Lipidomic Analysis Data Presentation for Halal Authentication of Meat Products

**DOI:** 10.3390/molecules27217571

**Published:** 2022-11-04

**Authors:** Vevi Maritha, Putri Widyanti Harlina, Ida Musfiroh, Amirah Mohd Gazzali, Muchtaridi Muchtaridi

**Affiliations:** 1 Department of Pharmaceutical Analysis and Medicinal Chemistry, Faculty of Pharmacy, Universitas Padjadjaran, Bandung 45363, Indonesia; 2 Department of Food Industrial Technology, Faculty of Agro-Industrial Technology, Universitas Padjadjaran, Bandung 45363, Indonesia; 3 School of Pharmaceutical Sciences, Universiti Sains Malaysia, USM, Penang 11800, Malaysia

**Keywords:** meat products, halal, metabolomic, lipidomic, chemometrics

## Abstract

The halal status of meat products is an important factor being considered by many parties, especially Muslims. Analytical methods that have good specificity for the authentication of halal meat products are important as quality assurance to consumers. Metabolomic and lipidomic are two useful strategies in distinguishing halal and non-halal meat. Metabolomic and lipidomic analysis produce a large amount of data, thus chemometrics are needed to interpret and simplify the analytical data to ease understanding. This review explored the published literature indexed in PubMed, Scopus, and Google Scholar on the application of chemometrics as a tool in handling the large amount of data generated from metabolomic and lipidomic studies specifically in the halal authentication of meat products. The type of chemometric methods used is described and the efficiency of time in distinguishing the halal and non-halal meat products using chemometrics methods such as PCA, HCA, PLS-DA, and OPLS-DA is discussed.

## 1. Introduction

The halal status of meat products is an important factor being considered by many parties, especially Muslims. As meat is an essential source of high-quality protein, essential amino acids, vitamins, and minerals [1,2], the science and knowledge pertaining to halal food continues to expand in multiple directions over the years [3]. In general, the halal status of a meat product is determined by several factors; originating from halal meat (such as poultry, lamb, and beef) and the slaughter process is accomplished in accordance with the Sharia [4]. Non-halal meats, on the other hand, include those originated from pork, rats, and dogs, and non-halal compounds such as lard or pork fat. Authenticating the halal status of meat products is crucial and has to be performed using the correct analytical methods, especially since the analysis of products that have undergone extensive processing is rather challenging [5]. Indeed, a good and highly specific analytical method is needed to enable the detection and separation between halal and non-halal meat sources [6].

Among the methods that have good specificity and are applicable for this purpose are metabolomic and lipidomic analysis. Metabolomic is a method able to identify metabolites in small molecular sizes [7], while lipidomic is an analytical method that could identify lipids and sub lipids [8]. Metabolites and lipids are two types of compounds that are specific and unique to each animal species [9,10]. These two are able to identify metabolites (metabolomics) and lipid and sub lipids (lipidomic) from non-halal meat, meat that is sourced from non-Sharia slaughter processes, and non-halal components [11,12]. In both of these, a large amount of data is produced, so chemometrics are needed to simplify the data presentation [13].

Chemometrics are analytical methods that combine chemical and statistical data to visualize, group, and classify samples [14]. These methods can provide clear visualization for halal and non-halal products [15]. In halal meat products analysis, chemometrics can be the determinants of halal status, as they can be used to classify and separate halal from non-halal meat products [16].

This review will discuss the chemometrics methods that can be applied for metabolomic and lipidomic studies in the halal authentication of meat products. The information gathered and presented in this review will be useful to guide researchers involved in halal product authentication for effective data management and analysis to ensure accurate determination of halal status, specifically for meat-based products [17].

## 2. Halal Meat Products

Consuming halal meat products is an obligation for Muslims, but they are also accepted by non-Muslims [18]. Along with the increasing number of Muslims [19,20] and the high consumption of meat-based products [21], many parties have started to pay special attention to the authentication of the halal status of meat products sold or served to customers [22]. Consumer sensitivity has led to the expansion of the halal meat products market around the world [23]. This situation has pushed meat products producers to pay close attention to the quality of their meat products to ensure good market acceptance. With respect to the production of halal meat, there are several rules and regulations that need to be met by meat producers. Among others, according to the Sharia, slaughtering of feedstock animals must be performed in a suitable manner so as to prevent excessive pain to the slaughtered animals [24]. This practice gives the connotation that halal meat products are of good quality, clean, safe [25], and healthy for consumption [26,27]. In addition, the regulation of halal status is indeed important to assure the quality and standard of halal meat products [28]. This can be accomplished through an official certification and placement of an official logo on the products as a mean to provide security to consumers [28,29,30,31,32,33,34,35].

## 3. Chemometrics

Chemometrics is a science that combines chemistry with statistics to simplify data presentation [36,37,38]. The application of chemometrics in managing chemical-related data has been growing [39] due to its strength in data solving such as in metabolomic and lipidomic analyses [40,41]. It allows controlling the number of variables involved in the analysis [42,43] and providing accurate and significant results in a short time [44], besides having a good sensitivity and robustness [45]. There are two general types of chemometric methods, the unsupervised classifications and supervised classifications [46]. Unsupervised classifications include hierarchical clustering analysis (HCA) or clustering analysis. Supervised classifications, on the other hand, include linear discriminant analysis (LDA), support vector machine (SVM), partial least square discriminant analysis (PLS-DA), orthogonal projection to latent structure-discriminant analysis (OPLS-DA), counter propagation artificial neural networks (CP-ANNs), self-organizing maps (SOMs), and random forests (RF) [47]. Of the various chemometric methods, only a few are frequently applied to metabolomic and lipidomic analyses, such as PCA, clustering analysis, and LDA [48].

PCA is a statistical technique used to simplify a large amount of data without compromising the main information [49]. The use of PCA in metabolomic and lipidomic analyses is useful to manage LCMS data analysis, to enable the detection of compounds of interest, and to the detect the presence of meat from other animal species [50,51,52,53]. Cluster analysis is used to divide groups based on their equations [54,55]. In metabolomic or lipidomic analyses, this method is used to group samples based on predetermined metabolites or lipids [56], according to the statistical analysis conducted [57]. LDA, on the other hand, is able to distinguish different types of meat based on their metabolite or lipid profiles [58], such as differentiating between domestic pork species based on their lipid profiles [59].

**Figure 1:**
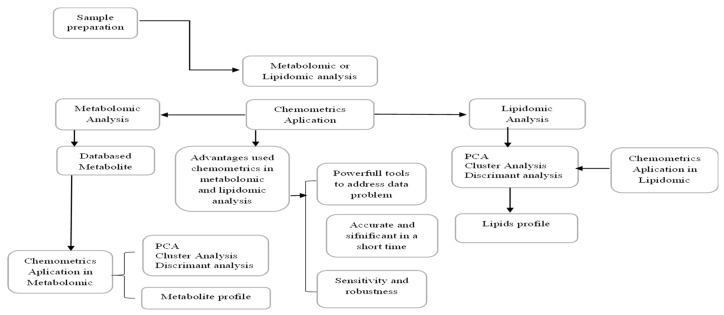
Chemometric applications in metabolomic and lipidomic studies.

The application of chemometrics to metabolomic and lipidomic analyses can be used to focus on the preprocessing and variable selection methods [60]. A detailed examination of preprocessing methods for a given data set is critical as these methods can also remove relevant chemical information. Therefore, the search for the best preprocessing method is vital, considering its impact on the subsequently performed data analysis and its outcome. These preprocessing methods can be employed to either remove noise contributions, replace missing values, interpret or remove baselines, or even a combination of these targets [61,62,63]. Variable selection methods, on the other hand, can guide the choice of method in practical data analysis [64]. Variable selection methods, such as variable important projection (VIP), selectivity ratio (SR), and significance multivariate correlation (sMC), were also applied to select the most effective wavelengths in the analysis of metabolomic and lipidomic studies using spectroscopy [65]. Figure 1 illustrated the different chemometric applications for metabolomic and lipidomic analysis.

## 4. Chemometrics Applications in Metabolomic Studies for Halal Authentication of Meat Products

Metabolomic analysis is the study of metabolites with small molecular weight (˂15,000 Da), which are usually the end products of cellular metabolism [66,67,68,69,70,71]. Metabolomics have the ability to analyze comprehensively the overall metabolites contained in meat and meat products, including mixes of non-halal meat, oil, or other non-halal compounds, and differentiating the meat that has or has not undergone a Sharia-compliant slaughter process [72,73]. In the case of mixing meat in meat products, it is difficult to distinguish the type of meat contained in the product. The difference in the metabolites between various meat samples can be used as a reference to overcome this problem, so the metabolomic approach may help to solve this issue. To analyze metabolomic data, chemometric methods will hence be used [74].

Chemometric analysis is used to design, process, visualize, explore, and analyze metabolomic data [75]. The most common data analysis method employed in authenticating halal in meat products using chemometrics is multivariate analysis [76]. Multivariate analysis in metabolomics is useful in the analysis of data that contain complex variables in the authentication of halal meat products [77]. The popular methods include PCA, PLS-DA, and OPLS-DA, which are capable of distinguishing, grouping, and classifying meats such as pork, chicken, and beef [78]. PCA is the most widely used method in which it reduces the number of metabolites to the main metabolite to determine the halalness of the product [79]. From LC-MS results, PCA could detect other animal species in a sample [80]. Classification is a part of multivariate analysis that could distinguish halal and non-halal meat products through the PLS-DA method. PLS-DA could analyze highly collinear and noisy data [81] especially if the number of variables exceeds the number of samples [82]. Among the reported applications of PLS-DA in metabolomic analysis was the authentication of halal chicken meat according to the method of slaughter. FTIR spectrum showed slightly different metabolite fingerprints of the two meats. This was confirmed due to the presence of different metabolites in the NS (neck slaughtering) and NP (neck pocking) chicken meat, which was further validated by differences in the GC-MS and UHPLC-TOF-MS data following PCA and PLS-DA analysis. In comparison to NP, NS chicken meat is rich in metabolites with health benefits, including *N*-3-polyunsaturated fatty acids (PUFA), triglycerides (TG), cytidine, and uridine. In addition, NS chicken meat also contains significantly lower concentrations of free amino acids [83].

OPLS-DA is another fast, simple, and efficient multivariate analysis method. It was applied to the screening of beef specific quantitative peptides based on liquid chromatography tandem mass spectrometry (LC-MS/MS). The OPLS-DA model was created to select species-specific peptides that contribute significantly to classification. Peptides with statistical significance were selected based on significant variables in the projected value (VIP) and the univariate P value. After a statistical process workflow, three specific quantitative peptides were identified using homemade products with different beef contents. The quantitative results were then applied to commercial beef products that confirmed the high sensitivity, specificity, and repeatability of the developed method. The results of this study proved the integration of LC-MS/MS combined with OPLS-DA as an efficient method for screening certain quantitative peptides and authenticating halal meat products with selected peptides as markers.

Ali et al. [84] investigated that score plots obtained from PCA supported to elucidate the differences and similarities between the metabolic profiles of halal and non-halal chickens. A similarity map obtained by applying PCA to the UHPLC-TOF-MS spectra showed that PC1 and PC2 accounted for 65.8% of the total variance. Furthermore, classification of metabolite profiles of halal and non-halal chickens showed that PC1 and PC2, respectively, predicted for 44.6% and 21.2% of the total variance. In this study, PLS-DA is used to distinguish between halal and non-halal chickens. PLS-DA can predict metabolites that determine broiler health based on the slaughtering technique. The result showed that non-halal chicken had higher concentration of inosine and histidine while halal chicken showed higher concentration of hypoxanthine. Table 1 summarized the literature on the application of chemometric methods in metabolomic analysis for the halal authentication of meat products.

## 5. Chemometrics Applications in Lipidomic Analysis for Halal Authentication of Meat Products

Lipidomic is a strategy that describes comprehensive lipid profiling [91]. Lipid is one of the most important organic compounds in living organisms, whereby it functions as cell membrane defense, energy storage, and signaling molecule [92]. Lipid is also species-specific, so that these differences in lipid profiles can be used for meat authentication [93]. In lipidomic analysis, both lipid and sub lipid can be detected, which has made it an attractive approach to authenticate halal meat products [94]. As an example, lipidomic analysis is able to distinguish halal and non-halal oil in a mix, based on the analysis of the lipid and sub lipid profiles, and to detect the presence of mixed meats and non-halal oils in meat products based on their lipid profiles [95,96]. The lipidomic method is able to distinguish between halal and non-halal meat fatty acids. For example, when there is a mixing of halal and non-halal oils, it is difficult to distinguish between the two so that the lipid and sub lipid profiles of the oils can be a determinant. Therefore, a lipidomic approach can solve this. In lipidomic analysis, a chemometric method is needed, namely multivariate analysis, to interpret the data obtained from spectra and chromatograms [97].

**Figure 2:**
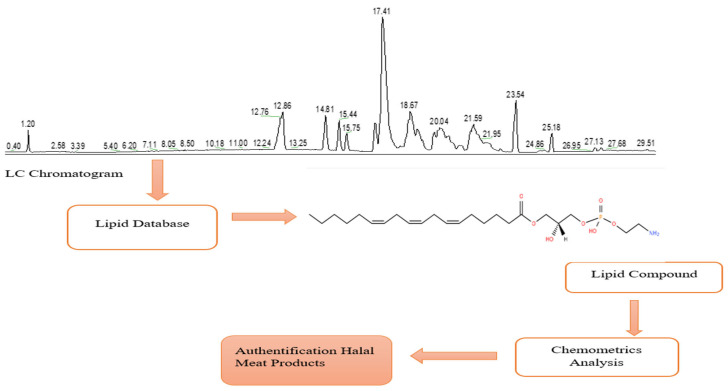
Data presentation of lipidomic study.

Multivariate analyses that are often applied to lipidomic analysis for the halal authentication of meat products are PCA, HCA, and PLS-DA [98]. PCA has the advantage of reducing data practically and being able to identify large variables [99]. In halal authentication using the lipidomic method, hundreds of lipid compounds will be detected in the LC-MS chromatogram with several replications, and PCA will be able to reduce them to the main lipids contained in the sample based on the equations generated in the chromatogram area. PCA score plots can explain the differences between halal and non-halal meat based on lipid compounds [100]. HCA, or cluster analysis, is used to group samples based on equations. HCA is able to classify halal and non-halal meat products based on the similarities between their lipid compounds [101], whilst discriminant analysis such as PLS-DA will distinguish the components present in the samples [102,103]. Combination of more than one chemometric method is commonly used in lipidomic studies to ensure accurate analysis and data interpretation [104]. Figure 2 is a summarized data presentation of a lipidomic analysis. Table 2 summarized the literature on the application of chemometrics in lipidomic analysis for the halal authentication of meat products.

For the halal authentication of meat products using lipidomic information, quite a lot of data are produced, so that the chemometrics methods used are not only single but also in combination to present information that is easier to understand [108]. PCA is a chemometric method that is most often combined with HCA or PLS-DA. This is accomplished to convey conclusions in the lipidomic analysis for the halal authentication of meat products. The first conclusion in this analysis is that the lipid or sub lipid component is the determinant of halalness, and this can be obtained with PCA and PLS-DA analysis [109]. The second conclusion in this analysis is which samples are included in the halal and non-halal groups, which can be obtained with PCA and HCA analyses [110]. The use of chemometric methods in lipidomic analysis is able to provide valid conclusions even though the data analyzed are very large.

**Figure 3:**
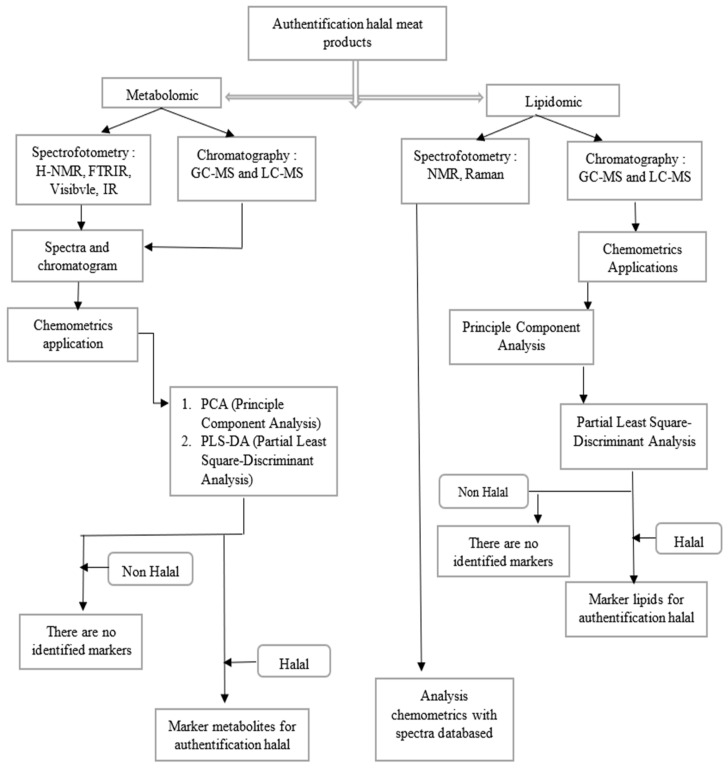
Chemometrics applications in metabolomic and lipidomic studies for halal authentication in meat products.

Trivedi et al. [107] performed a lipidomic analysis for the halal authentication of meat products, in which it was reported that PCA could be used to differentiate sham beef and pork at various concentrations. PCA from GC-MS data showed a clear gradient profile of increased amount of pork adulteration in the beef samples. This indicates that these data could provide quantitative information, as PC1 typically accounts for 50% in the four PCA score plots. In this report, PLS-DA analysis was also carried out which was able to explain the types of significant fat found in a mixture of beef and pork, including TG (16:0/15:0/18:4), Cer (d18:1/24:1), CE (22:5), and TG (16:0/15:0/18:4) [107]. Taylan et al. (2020) showed that, as evidenced from the HCA dendrogram, butter fat samples *(n* = 3) were clearly distinguished from adulterated and lard fat samples. Additionally, lard fat samples were distinguished from butter fat and adulterated samples in the left arm of the HCA dendrogram. A well-separated cluster with a high heterogeneity score of 400 was observed. The examined samples were grouped into two His grades, primarily numbered ‘1’ and ‘2’. Arms numbered ‘1’ were split into two clusters. These subclusters were numbered ‘3’ and ‘4’. Adult samples with the highest foreign body contamination rate (40%) were clustered separately on the arm numbered ‘3’. Therefore, butterfat was clustered separately from lard and adulterated samples with different lard content (3%, 5%, 10%, 20%, and 40% *w*/*w*). It can be interpreted from the HCA dendrogram that butterfat can be distinguished from lard and adulterated samples with high-quality visualization of interrelationships between clusters and subclusters [105]. Figure 3 provides a summary of the applications of chemometrics in metabolomic and lipidomic studies for the halal authentication of meat products.

## 6. Methods

**Figure 4:**
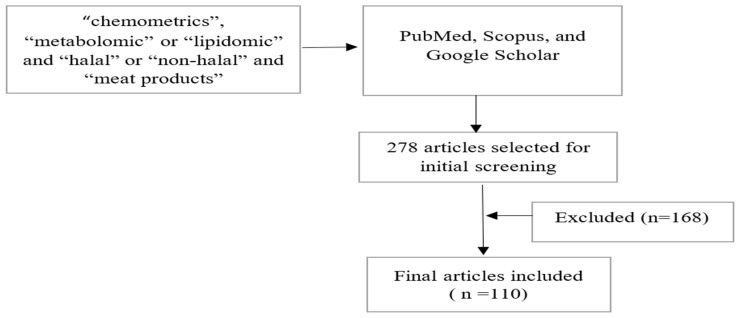
Flowchart of methodology.

PubMed, Scopus, and Google Scholar databases were searched with the following “chemometrics”, “metabolomic” or “lipidomic”, “halal” or “non-halal”, and “meat products”. The relevance of the articles obtained with the reviewed topic were then determined. The number of articles that matched the keywords was 278 for the range 2012 to 2022, but 110 articles were used. This is because some did not use chemometric analysis in the manuscript, as well as lipidomic or metabolomic analysis instead of meat. The flowchart of the methodology is shown in Figure 4.

## 7. Conclusions and Future Perspective

Metabolomic and lipidomic methods are strategies that are specific in analyzing the presence of metabolites and lipids in living organisms. As metabolites and lipids are species-specific, they are highly suitable as markers to authenticate the halal status of meat products. They can also be used to determine whether a meat product is obtained through the Sharia-compliant slaughtering process or is mixed with non-halal materials. During metabolomic and lipidomic analyses, large amounts of data will be generated and thus a strong data analysis approach, such as a chemometric method, is necessary to interpret and present the data in a simplified and understandable manner. Among the frequently used chemometric methods employed in metabolomic and lipidomic studies are PCA, HCA, PLS-DA, and OPLS-DA, which are accurate, fast, and robust in processing data. The current available literature has described the ability of these chemometric methods to analyze, distinguish, and classify data presentation of halal and non-halal meat products using metabolomic and lipidomic analysis.

## Figures and Tables

**Table 1:** Chemometric applications in metabolomic analysis for halal authentication of meat products.

No	Title	Objectives	Equipment	Chemometrics Techniques	Results	Ref.
1	Volatilomics for halal and non halal meatball authentication using solid-phase microextraction-gas chromatography-mass spectrometry	Meatball	GC-MS	PLS-DA	PLS-DA was able to distinguish volatile compounds in samples	[85]
2	The Feasibility of Two Handheld Spectrometers for Meat Specification Combined with Chemometric Method and Its Application for Halal Certification	Meat (lamb, beef, chicken, pork)	(Vis-NIR) and (NIR) spectroscopy	PLS-DA	PLS-DA was able to classify meat types with an accuracy value of 88.3%	[73]
3	^1^H-NMR-Based Metabolomic: An Integrated Approach for the Detection of the Adulteration in Chicken, Chevon, Beef and Donkey Meat	Meat (chicken, chevon, beef and donkey)	^1^H-NMR	PCA and OPLS-DA	PCA was able to identify 37 metabolites while OPLS-DA was able to distinguish the types of chicken, chevon, beef, and donkey meats	[86]
4	Authenticity Analysis of Beef Meatball Adulteration With Wild Boar Using FTIR Spectroscopy Combined With Chemometrics	Beef meatball and wild boar	FTIR	PCA and PLS	PCA would differentiate wild boar meatball and beef meatball products. PLS gave the value of determination coefficient (R2) of 0.9991	[87]
5	Chemometrics-Assisted Shotgun Proteomics for Establishment of Potential Peptide Markers of Non-Halal Pork (Sus Scrofa) among Halal Beef and Chicken	Beef and chicken	LC-MS	PCA and OPLS-DA	PCA was able to reduce the data of metabolites that have similarities. OPLS-DA differentiated the results from PCA of beef and chicken based on the slaughter process	[88]
6	Discrimination between vegetable oil and animal fat by a metabolomics approach using gas chromatography-mass spectrometry combined with chemometrics	Lard	GC-MS	PCA	PCA was able to distinguish types of fat	[89]
7	Identification of Metabolomic Profile in Halal and Non-Halal Broiler Chicken Using Fourier-Transform Infrared Spectroscopy (FTIR) and Ultra High-Performance Liquid Chromatography-Time of Flight-Mass Spectrometry (UHPLC-TOF-MS)	Chicken broiler	FTIR and UHPLC-TOF-MS	PCA and PLS-DA	PCA was able to classify the metabolites found in broiler chickens based on the method of slaughter. PLS-DA distinguished non-halal chicken by the presence of high histidine and inosine	[84]
8	Untargeted-metabolomics different between poultry samples slaughtered with and without detaching spinal cord	Chicken meat	LC-ESI-MS/MS	PCA and OPLS-DA	PCA characterized metabolites based on the mode of slaughter. OPLS-DA was able to classify halal and non-halal samples	[90]

**Table 2:** Chemometric applications in lipidomic studies for halal authentication of meat products.

No	Title	Objectives	Equipment	Chemometrics Techniques	Results	Ref.
1	Detection of lard in butter using Raman spectroscopy combined with chemometrics	Lard	Spectroscopy Raman	HCA and PCA	HCA and PCA were successfully performed for the classification and discrimination of butter and lard-adulterated samples.	[105]
2	Liquid Chromatography Quadrupole Time-of-Flight Mass Spectrometry and Rapid Evaporative Ionization Mass Spectrometry Were Used to Develop a Lamb Authentication Method	Lamb	LC-QTOF-MS	PCA and OPLS-DA	PCA was performed to identify. OPLS-DA was carried out to separate the samples to the largest extent	[106]
3	The Metabolites: An Integrated metabolite Profiling and Lipidomic Approach for The Detection of The Adulteration of Beef With Pork. Analyst	Beef and pork	GC-MS	PCA and PLS-DA	PCA and PLS-DA was able to distinguish beef that contained pork	[107]
4	Multivariate Analysis Coupled with M-SVM Classification for Lard Adulteration Detection in Meat Mixtures of Beef, Lamb, and Chicken Using FTIR Spectroscopy	Lard	FTIR	PCA	PCA was able to classify with an accuracy value of 85%	[31]

## Data Availability

The data presented in this study are available on request from the corresponding author.
